# Wnt signaling in cancer stem cells and colon cancer metastasis

**DOI:** 10.12688/f1000research.7579.1

**Published:** 2016-04-19

**Authors:** Sayon Basu, Gal Haase, Avri Ben-Ze'ev

**Affiliations:** 1 Department of Molecular Cell Biology, Weizmann Institute of Science, Rehovot, Israel

**Keywords:** Wnt, colorectal cancer, intestinal stem cells, cancer stem cells, β-catenin, Lgr5

## Abstract

Overactivation of Wnt signaling is a hallmark of colorectal cancer (CRC). The Wnt pathway is a key regulator of both the early and the later, more invasive, stages of CRC development. In the normal intestine and colon, Wnt signaling controls the homeostasis of intestinal stem cells (ISCs) that fuel, via proliferation, upward movement of progeny cells from the crypt bottom toward the villus and differentiation into all cell types that constitute the intestine. Studies in recent years suggested that cancer stem cells (CSCs), similar to ISCs of the crypts, consist of a small subpopulation of the tumor and are responsible for the initiation and progression of the disease. Although various ISC signature genes were also identified as CRC markers and some of these genes were even demonstrated to have a direct functional role in CRC development, the origin of CSCs and their contribution to cancer progression is still debated. Here, we describe studies supporting a relationship between Wnt-regulated CSCs and the progression of CRC.

## Introduction

Wnt signaling has emerged during evolution as a highly conserved signaling pathway that regulates tissue morphogenesis and regeneration (via stem cells) in various tissues of multicellular organisms ^1^. Hyperactivation of β-catenin-T cell factor (TCF)/lymphoid enhancer factor (LEF)-regulated gene transcription (the end point of Wnt signaling) is a hallmark of colorectal cancer (CRC) development. β-catenin is also a key regulator of cell-cell adhesion, by linking the E-cadherin transmembrane adhesion receptor that binds neighboring epithelial cells to each other, to the actin-cytoskeleton ^2^. Therefore, activation of the Wnt pathway in CRC provides an attractive model for studying the links between tissue morphogenesis and cell adhesion and the disregulation of these processes during cancer progression.

The canonical Wnt pathway is also known as the Wnt-β-catenin pathway since β-catenin is a key transducer of the Wnt signal from the cytoplasm to the nucleus. In unstimulated cells, the free pool of β-catenin (the one not engaged in cadherin-mediated cell-cell adhesion) is phosphorylated by a complex of proteins that includes the scaffold molecule Axin and adenomatous polyposis coli (APC) and the kinases glycogen synthase kinase 3β (GSK3β) and casein kinase 1 (CK1) ^1, 2^. After phosphorylation, β-catenin is targeted for proteolytic degradation by the proteasome. Wnt is secreted from cells as a lipid-modified molecule that acts in short-range signaling ^3, 4^ and stimulates signaling by binding of the Wnt ligands to the Frizzled transmembrane receptors and to the Lrp5/6 co-receptors. The cytoplasmic tail of Lrp becomes phosphorylated, inhibits GSK3β, and associates with Axin. Wnt signaling is positively regulated by the secreted R-spondins that act to stabilize the Frizzled receptors against degradation by the Rnf43/Znrf3 ubiquitin ligases ^5, 6^. Activation of the Wnt pathway results in the disruption of the CK1-GSK3β-Axin-APC-β-catenin complex, inhibition of GSK3β activity, and the stabilization of β-catenin against degradation in the cytoplasm by the ubiquitin-proteasome pathway. The accumulation of β-catenin in the cytoplasm results in its nuclear translocation. In the nucleus, β-catenin binds to members of the TCF/LEF family of transcription factors and plays a role as a co-activator of target gene transcription ^7^. In CRC, aberrant activation of the Wnt signaling pathway is a central oncogenic driver in 90% of patients, mostly resulting from mutations in the *APC* gene ^8^. Expression of genes by the aberrant transcriptional activity of the β-catenin-TCF complex contributes to both the initial stages of the disease and the later stages involving invasion and metastasis ^9^. Here, we describe recent findings on the involvement of Wnt signaling in CRC progression and its relationship to the emerging role of cancer stem cells (CSCs) in CRC.

## Wnt signaling in intestinal stem cell homeostasis

Intestinal epithelial cells display the highest turnover rate, and the entire intestinal epithelial lining in humans is replaced every 5 to 7 days ^10^. This rapid regeneration is fueled by the proliferation of stem cells at the base of the intestinal crypts of Lieberkühn and the upward migration and differentiation of stem cells that enables normal tissue homeostasis. The morphological separation of the stem cell compartment (the crypt where the cells proliferate) and the differentiated compartment (villus in the intestine, and the surface epithelium in the colon, where the cells interact with the gut environment) depends on a gradient of Wnt signaling. The strongest Wnt signaling is detected at the crypt base (where some cells display nuclear β-catenin localization) and gradually weakens toward the luminal side of the vertical crypt-villus axis ^11^. Wnt signaling is necessary for the initial potentiation of intestinal stem cells (ISCs) as evident from studies in neonatal transgenic mice that lost TCF4 and thus fail to develop normal proliferative crypts ^12^. Both crypt homeostasis and stem cell maintenance require active Wnt signaling since conditional activation of Wnt antagonists in transgenic mice leads to the progressive loss of intestinal crypts ^13– 15^. Similarly, conditional abrogation of Wnt signaling in cells at the crypt base, by deletion of either β-catenin ^16^ or TCF4 ^17^, leads to the loss of proliferative crypts.

The intestinal crypt has long been recognized as the niche for proliferative, multipotent precursor cells of the intestine and colon, and the Wnt target gene *Lgr5*, a receptor for the Wnt agonist R-spondin that enhances Wnt signaling, was identified as a marker for columnar crypt base stem cells ^18^. Lineage-tracing experiments in transgenic mice revealed that Lgr5 ^+^ cells found in the crypt base are multipotent and capable of clonally repopulating the entire epithelial lining of the intestine and colon ^18^. Gene expression and proteomic signature studies of Lgr5 ^+^ cells revealed several additional ISC markers, including Ascl2 and Sox9 ^19^. The basic helix-loop-helix (bHLH) transcription factor Ascl2 is a major transcriptional regulator of genes associated with stemness in crypt cells and is a key ISC marker ^20^. Similarly, the transcription factor Sox9 is also expressed by stem cells in the intestinal and colonic crypt base and is necessary for the maintenance of ISCs ^21, 22^. Like *Lgr5*, *Ascl2* and *Sox9* are also Wnt target genes in ISCs. This points to the requirement for high Wnt signaling in the maintenance of the stem cell niche ^23^.

Experiments tracking cell proliferation and migration in the intestine identified as putative stem cells, cells at position +4 (4 cells up from the crypt base) in the intestine. These cells display proliferative regeneration in intestinal epithelia upon cytotoxic damage and are highly sensitive to radiation-induced apoptosis ^24, 25^. The cells at position +4 within the intestinal crypt undergo continuous proliferation while retaining ^3^H-labeled DNA (hence, the cells are named label-retaining cells, or LRCs) and support the notion that “+4” LRCs function as stem cells ^26^. Bmi1, a chromatin silencing component, was identified as a marker for LRCs, and lineage-tracing experiments revealed that Bmi1 ^+^ LRCs are undifferentiated stem-like cells. Bmi1 ^+^ LRCs may either self-renew or clonally expand and differentiate into all cell types of the intestinal mucosa, including Lgr5 ^+^ columnar crypt base cells ^27, 28^. Unlike the turnover rate of Lgr5 ^+^ crypt base stem cells, that of Bmi1 ^+^ LRCs (situated just above the crypt base) is much slower, indicating that they are probably not the major stem cell type that functions in intestinal homeostasis ^27^ and are proposed to function as reserve stem cells in response to tissue damage ^29^. In addition to Bmi1, Hopx, Tert and Lrig1 are also markers of LRCs ^30– 32^. Although Lgr5 and Bmi1 are apparently markers of two distinct subpopulations of stem cells, there is an overlap between these markers with Bmi1 being strongly expressed by a subset of Lgr5 ^+^ ISCs ^19^. Apart from LRCs, committed Dll1 ^+^ secretory progenitor cells located even further upwards from the crypt bottom also retain the ability to re-acquire stem cell functions and regenerate the stem cell compartment in response to tissue damage ^33^. Although strong Wnt signaling and the paracrine context at the crypt base are essential components that regulate the maintenance of the ISC pool, more differentiated cells retain sufficient plasticity that allows them to revert to a stem cell-like behavior under stressful conditions ^29, 30, 34^. Since genotoxic stress and other carcinogenic perturbations may affect the stem cell pool, they may also play a key role in the development of CRC ^34^.

## Cancer stem cells and Wnt signaling in colorectal cancer

CSCs are hypothesized to constitute a small fraction of the tumor tissue. In a role similar to that of ISCs in normal tissue, CSCs are suggested to give rise to progenitors that populate the majority of the tumor ^35^. Two models describing the histogenesis of CRC have been proposed: the ‘‘top-down’’ and the “bottom-up” morphogenesis. The “top-down” model suggests that the more differentiated (luminal) cells re-acquire stem cell-like properties and produce aberrant crypt foci where tumors develop ^36^. The ‘‘bottom-up’’ histogenesis suggests that stem cells residing at the crypt base expand and migrate upwards and constitute the tumor-initiating cells ^37^. In both models, Wnt signaling is considered an important regulator. According to the “top-down” theory, hyperactivation of β-catenin-TCF signaling drives differentiated epithelial cells into regaining pluripotency, thereby forming new, disregulated crypt-like structures that later turn into adenomas ^38^. The increased frequency at which very early adenomatous polyps are observed at the top of colonic crypts (far removed from the stem cell compartment) has led researchers to suggest that neoplastic transformation in CRC is initiated from differentiated cells ^39^. Other studies in transgenic mouse models for intestinal cancer have shown that differentiated epithelial cells can re-acquire stem cell-like properties upon the combined activation of Wnt and nuclear factor-κB (NF-κB) signaling, conferring tumor-initiating cell properties ^38^. On the other hand, immunohistochemical studies of early sporadic colorectal adenomas have shown adenomatous lesions near the crypt base ^37^. These lesions have increased proliferative activity with nuclear β-catenin localization while their corresponding surface epithelial cells maintain β-catenin in sub-membrane adherens junctions (not in the nucleus) ^37^. Moreover, the conditional loss of APC in Lgr5 ^+^ colonic crypt base stem cells induced their rapid transformation into micro-adenomas, indicating that increased Wnt activity in the Lgr5 ^+^ stem cell compartment may trigger a tumor-initiating process ^40^. Similarly, conditional activation of β-catenin in Bmi1 ^+^ LRCs led to an immediate generation of adenomas in the duodenum ^27^. These studies support the “bottom up” histogenesis of CRC suggesting that excessive Wnt activation in the stem cell compartment is an essential step in neoplastic transformation. Chronic inflammation and other conditions that increase NF-κB signaling support the notion that a dedifferentiation step can occur in intestinal epithelia, supporting CRC development by a “top down” histogenesis. Conversely, if stem cells at the crypt bottom acquire mutations that lead to activation of Wnt signaling, CRC may arise by the “bottom up” model.

One way or the other, the overriding importance of Wnt signaling in CRC development, as compared with other driving oncogenes in CRC, such as *Kras* and *p53*, was recently demonstrated by an effective, conditional suppression of APC by using small hairpin RNA (shRNA) in transgenic mice ^41^. The suppression of APC by this method resulted in intestinal and colon cancer development in mice. Restoration of APC expression in these tumors resulted in the reversal of tumorigenic lesions and the complete reconstitution of a normal stem cell compartment, even in mice harboring oncogenic *Kras* and *p53* ^41^.

In CRC tissue, Wnt signaling (as gauged by nuclear β-catenin localization) is not homogenous, even though all CRC cells harbor an activating mutation in the Wnt pathway ^42^. This observation, called “the β-catenin paradox”, was first observed by immunohistochemical analyses of CRC tissue. Well-differentiated cells, located in the more central areas of the tumor, display mostly β-catenin associated with E-cadherin under the membrane, as in the normal intestinal epithelium, whereas tumor cells localized at the invasive front of the same tumor exhibit strong nuclear β-catenin staining ^43^. These invasive edge-localized cells were shown to preferentially express Wnt target genes that confer invasive-metastatic capacity when expressed in human CRC cells ^44– 46^. Vermeulen *et al*. ^47^ investigated the role of Wnt signaling in the homeostasis of CSCs in human CRC: CSCs isolated from patients and cultured as spheroids displayed a heterogeneous level of Wnt signaling over a 100-fold change. The Wnt ^high^ cells formed more tumors in mice with fewer injected cells compared with Wnt ^low^ cells. This heterogeneity in Wnt signaling was maintained in the tumors formed in mice that also expressed several ISC markers, including Lgr5 and Ascl2 ^47^. The importance of Wnt signaling in the maintenance of the CSC pool and in driving CRC progression is also highlighted in a study in which conditional activation of the Wnt antagonist HoxA5 suppressed tumor growth and metastatic progression by repressing stemness properties ^15^.

## Lgr5 as a Wnt-induced cancer stem cell marker

Lgr5, a target gene of Wnt signaling, is a well-established ISC marker ^18^. Studies involving mouse models of intestinal cancer have provided the initial evidence that Lgr5 ^+^ cells act as tumor-initiating cells, since activation of Wnt signaling (by conditional deletions of APC) in the Lgr5 ^+^ subpopulation of intestinal cells led to adenoma formation ^40, 48^. Recent studies attempting to define an ISC gene signature in CRC tissue repeatedly detected Lgr5 as a key component in such signatures ^49– 52^. The presence of Lgr5 on the surface of cells is sufficient for successful isolation of the CSC fraction from CRC tissue, and similar to its role in the normal intestine, Lgr5 defines the undifferentiated stem cell state in CSCs. CRC cells with high Lgr5 expression had enhanced ability to clonally expand and give rise to colonies *in vitro*, whereas suppression of Lgr5 expression results in the loss of their ability to form colonies ^51^. An ISC gene signature derived from EphB2 ^high^ columnar crypt base cells was suggested as a powerful predicting tool of human CRC progression and disease relapse ^50^. The EphB2 ^high^ ISC signature had a significant overlap with a previously described Lgr5-ISC signature ^19^. Tumors with high levels of Lgr5-ISC signature genes were more aggressive and metastatic and also displayed an increased tendency to relapse in patients with CRC ^50^. In an evaluation of 19 putative stem cell markers, Lgr5 was prevalently expressed in 74% of human CRC samples. Lgr5 and Ascl2 were significantly co-expressed with each other and with other genes from the list, supporting the hypothesis that adenocarcinomas are derived from Lgr5 ^+^/Ascl2 ^+^ crypt stem cells ^53^. However, other studies have defined a non-Wnt-induced CSC gene list that does not include *Lgr5* or other well-established Wnt-target genes. In a study on disease recurrence in CRC patients who went through curative surgery, a reverse correlation between Wnt-target genes ( *Lgr5*, *Ascl2*, *Axin2*, *Dkk1*, and *Apcdd1*) levels and disease recurrence was found, suggesting that elevated expression of Wnt-target genes is associated with good prognosis ^54^. Based on studies with CRC cell lines, these Wnt-target genes were silenced by CpG island methylation, and once methylation was inhibited, these cells lost their ability to generate colonies *in vitro*. These studies suggest that methylation of Wnt-target genes in CSCs is a strong predictor of CRC recurrence ^54^.

## The involvement of Wnt-induced cancer stem cells in colorectal cancer metastasis

According to the “β-catenin paradox”, only cells at the invasive front of the tumor tissue display strong nuclear β-catenin localization. These cells apparently go through an epithelial-to-mesenchymal transition (EMT), thus making them more motile and invasive, implying a role for the Wnt-induced CSCs in the propagation of metastasis. EMT has been suggested for some time as a key mechanism governing the generation of CSCs, especially as revealed by studies using breast cancer cell lines ^55^. In CRC, the EMT program influences a variety of malignant phenotypes associated with metastasis, including the generation of CSCs, tumor budding, circulating tumor cells, and drug resistance ^56^. The role of EMT in epithelial cancer, however, is still incompletely understood. Recent reports on lung and pancreatic cancer found that although EMT affects chemoresistance, it is not required for metastasis ^57, 58^. Since CSCs can both self-renew and differentiate, such cells can better adjust to the changes involved in the various stages of cancer metastasis ^59^. The involvement of CSCs derived by activation of Wnt signaling in the later stages of cancer progression was suggested in a study showing that Lgr5 expression correlated with the malignant potential of CRC tumors and cell lines ^49^. Tumors displaying increased levels of Lgr5 were of higher stage and were more invasive and formed more lymph node metastases ^49^. With the increasing number of studies suggesting the involvement of CSCs in the propagation of metastasis, the hypothesis of “migrating cancer stem cells” (MCSCs) was put forward as the driving force leading to metastasis ^59, 60^. According to this model, the inherent plasticity of CSCs is employed during the advanced stages of cancer progression that require the acquisition of invasive properties and migration through the blood and lymph vessels to distant organs. Newly formed metastatic tumors were shown to have a high genomic and proteomic similarity to the primary tumors from which they were derived, suggesting that after colonization MCSCs revert to their initial phenotype ^61^. A study comparing gene expression from primary CRC tissue and liver metastatic foci of the same patients found that the expression of *Ascl2* (an ISC marker and Wnt target gene) is upregulated in the metastatic tumors together with several other Wnt-induced ISC markers, including *Lgr5*, *EphB3*, *Ets2*, and *Sox9* ^61^. Other ISC signature markers, such as *Smoc2* ^19^, were also found to play a role in the promotion of metastasis in human CRC cells ^62^. Smoc2 expression was preferentially increased at the invasive edge of CRC tumors, and Smoc2 exclusively localized at the bottom of colonic crypts in the normal colonic epithelium ^63^. Moreover, L1-induced metastatic CRC cell lines lost their metastatic potential when Smoc2 was silenced ^62^.

## Crosstalk between Wnt signaling and other pathways in “stemness” and colorectal cancer metastasis

Although Wnt/β-catenin-TCF activation can directly influence the expression of “stemness” signature genes in CRC cells, such as *Lgr5* and *Ascl2*, Wnt signaling often interacts with other pathways in triggering the acquisition of a stem cell-like behavior in CRC cells. Inflammation-related signaling has long been implied as a key regulator in Wnt-β-catenin-driven cancers, including CRC ^63^. NF-κB signaling that regulates the pro-inflammatory cytokine response emerged as a key pathway in regulating the development of various cancers by being a potent driver of oncogenic signaling ^64^. In view of the important role of Wnt signaling in the maintenance of the stem niche in intestinal tissue and its deregulation in CRC, it was of interest to determine whether Wnt and NF-κB signaling interact in promoting CRC progression. This was recently addressed in the context of the cell adhesion receptor L1 (or L1CAM), a β-catenin-TCF target gene in CRC cells ^45^. L1 that is exclusively expressed in cells at the invasive edge of CRC tissue displaying nuclear β-catenin was found to activate NF-κB signaling by a mechanism involving the cytoskeletal protein ezrin ^65^. L1 and ezrin, together with IκB, form a complex that induces a more rapid degradation of IκB, followed by nuclear translocation and activation of NF-κB target genes ^66^. By blocking this L1-ezrin-NF-κB signaling, the acquisition of increased motility and liver metastasis by CRC cells was inhibited ^65^. L1-induced NF-κB activation leads to the expression of several ISC markers, including IGFBP2 ^66^ and Smoc2 ^62^. In another study, the loss of APC in CRC was shown to trigger the expression of a Rac1 GTPase, a member of the RACGEF family, via β-catenin-TCF-induced expression of the oncogenic transcription factor c-Myc ^67, 68^. As in the case of L1 ^66^, the activation of Rac1 leads to enhanced NF-κB signaling, resulting in the expansion of the Lgr5 ^+^ CRC stem cell compartment in an APC-deficient milieu ^68^. Thus, activation of NF-κB signaling in a Wnt ^high^ context may potentiate tumor cells to acquire a stem cell-like phenotype ^68^. In addition, a constitutive activation of β-catenin signaling in differentiated intestinal epithelial cells of transgenic mice was shown to trigger the expansion of intestinal crypt cells and requires the activation of NF-κB signaling ^38^. NF-κB was shown to directly bind to β-catenin and modulate its transcriptional activity, thereby affecting the expression of ISC signature genes ^38^. Lastly, the inflammatory microenvironment displaying highly active NF-κB signaling was shown to lead to the acquisition of a stem cell-like behavior and neoplastic transformation ^69, 70^.

Bone morphogenetic protein (BMP) signaling, through the transcriptional co-activator SMAD4, also plays an important role in CRC tumorigenesis ^71^. Mutations in the BMP receptor *BMPR1A*, or in *SMAD4*, underlie the juvenile polyposis syndrome, a rare autosomal dominant trait with increased risk for CRC ^71^. *SMAD4* mutations were shown to account for the shift in CRC tumor phenotype from the large adenoma to the adenocarcinoma stage ^72, 73^. Wnt-β-catenin signaling was reported to be required for BMP4 expression in CRC tumors ^74, 75^, and the transcription factor GATA6 affects both BMP and Wnt signaling in CRC stem cells ^76^. This is achieved by abrogating Wnt-triggered BMP4 expression in stem cells derived from colorectal adenoma that is apparently required for stem cell self-renewal in colon adenoma ^76^. Thus, the modulation (by the Wnt pathway) of the strength of NF-κB signaling, or of BMP and additional signaling pathways, is an important determinant of CRC progression. The strength of BMP signaling and its downstream messengers, including SMAD4, and additional driver mutations of CRC, such as p53, may affect the outcome of Wnt signaling in CRC development ^77^.

In addition to interacting with NF-κB and BMP signaling, the Wnt pathway affects other signaling molecules that are required for the acquisition and maintenance of the stem-like state in CRC cells. The β-catenin-TCF complex was shown to regulate the energy metabolism in CRC stem cells and to fuel the growth of CRC tumors by inducing the expression of the transcription factor PROX1 ^78, 79^. The cell adhesion receptor L1 (see above) induces the expression of the ISC marker *Clusterin* in CRC cells via STAT1 activation that is known to be stimulated by pro-inflammatory cytokines ^80^. The increased expression of the ISC marker *Msi1* ^81^, an RNA-binding protein and a β-catenin-TCF target gene, was linked to the elevated metastatic potential and poorer prognosis of CRC ^82, 83^. Msi1 can trigger the activation of Wnt and Notch signaling by a positive feedback regulation in ISCs, a regulatory loop recapitulated during CRC development ^84^. The *Msi1* homolog, *Msi2* (also a β-catenin-TCF target gene), displays an increased expression in intestinal cancer and drives the proliferation of stem-like cells through inhibition of PTEN and by inducing the mTORC1 pathway ^85^. Thus, together, these results suggest that the increase in Wnt signaling, even in more differentiated CRC cells, promotes the acquisition of a phenotype resembling that of ISCs by reconstituting a signaling environment that supports dedifferentiation.

## Conclusions

A microenvironment enabling high Wnt signaling supports stem cell renewal at the base of the intestinal crypts of Lieberkühn and apparently leads to the acquisition of stem cell-like properties in cells at the invasive edge of CRC tissue. Although a link between “stemness” properties and metastasis was suggested by numerous studies, the existence of CSCs has been difficult to identify in clinical tumor samples ^35, 86^. A common concept in cancer development suggests that tumors arise from proliferation and survival of a clonal subpopulation of stem cells within the tumor. However, studies with CRC indicate that tumors may arise from several different parent cells, each contributing a distinct lesion and thus generating polyclonal tumors ^87^. A polyclonal adenoma was described in an XO/XY individual with familial adenomatous polyposis (FAP) ^88^. Polyclonal adenomas were also observed in mice with a chimeric loss in APC in the intestinal epithelium ^89^. Conditional deletion of APC in stem cells labeled with a fluorescence reporter for Lgr5 triggered the development of adenomas from different cell clones within the intestinal tract ^48^. Given the high plasticity of the intestinal and colonic crypt cells and their ability to readily revert to “stemness” upon stress, it is possible that the CSCs in CRC tissue originate from different lineages of parent cells. Although a stem cell hierarchy is supposed to exist in the CRC tissue, the high plasticity also means that the expression of ISC signature genes may be heterogeneous and thus cancer cells not originating from ISCs may also express ISC signature genes to some extent. Thus, CRC cells may express various stem cell markers without re-acquiring a full “stemness” potential. The contradictory reports regarding the association of Lgr5 with various stages of CRC progression ^49, 50, 53, 54, 90– 92^ could be explained by the heterogeneity among the cells of the CRC tissue, as related to the expression of stem cell markers. Our current understanding of stem markers comes from studies on Wnt target genes or of markers of the Bmi1 ^+^ LRCs that were identified as putative cells of the stem cell compartment. Given that some stem cell markers are not dependent on Wnt signaling, further studies are required to determine the functional relevance of the many genes identified as stem cell signature genes in both normal and CRC tissue. Determining their roles in CRC not only will provide a better understanding of their function in intestinal homeostasis but will provide novel markers for targeting CRC. Using expression profiles for multiple stem cell markers in tandem increases the successful prediction of prognosis and outcome in CRC ^50^. Further studies of the stem cell niche and the molecules controlling self-renewal will provide a better definition of markers for the stem cell compartment. Current paradigms propose that treatments against cancer that fail to eradicate the CSC population will have little success in preventing future relapses of the disease. If correct, this hypothesis calls for additional research aiming to identify and understand the role of the subpopulations of CSCs in cancer development and for their more effective targeting.

## Abbreviations

APC, adenomatous polyposis coli; BMP, bone morphogenetic protein; CK1, casein kinase 1; CRC, colorectal cancer; CSC, cancer stem cell; EMT, epithelial-to-mesenchymal transition; GSK3β, glycogen synthase kinase 3β; ISC, intestinal stem cell; LEF, lymphoid enhancer factor; LRC, label-retaining cell; MCSC, migrating cancer stem cell; NF-κB, nuclear factor kappa light chain enhancer of B cells; TCF, T-cell factor.
